# The Effect of Selected Parameters of Vibro-Abrasive Processing on the Surface Quality of Products Made of 6082 Aluminium Alloy

**DOI:** 10.3390/ma12244117

**Published:** 2019-12-09

**Authors:** Marcin Stańczyk, Tomasz Figlus

**Affiliations:** Faculty of Transport and Aviation Engineering, Silesian University of Technology, 40-019 Katowice, Poland; tomasz.figlus@polsl.pl

**Keywords:** vibro-abrasive processing, surface treatment, grinding, deburring, surface roughness, aluminium alloy 6082

## Abstract

Vibro-abrasive processing is the basic method for the mass finishing of parts and components in various industries. Continuous progress in the development of processing media and machine design solutions means that every research effort into vibro-abrasive processing broadens the scope of knowledge in the selection of media, parameters, and applications in various industry fields. In this paper, an attempt is made to parametrize the vibratory grinding process, which is one of the three stages of high gloss finishing. Samples of the 6082 aluminium alloy intended for use in loaded machine parts and forged car wheel rims were subject to a research analysis. The samples were processed in a rotary vibrator equipped with a sample fixing system, using resin media and auxiliary com-pounds. On the basis of the analysis, the processing capacities were determined for the selected conditions and abrasive media. The influence of time and applied processing media on the change in samples’ roughness was determined. The effects of processing were examined with the use of laser devices measuring surface roughness in the areas of 3D and 2D analysis. The analysis of the test results showed that the use of S12TZ type resin media in a 12-hour finishing process of the 6082 aluminium alloy allowed for a 75.5% reduction in surface roughness, which corresponds to approximately 6.3% per hour of processing.

## 1. Introduction

Technological development and progress in the automation of processing methods is the basis for highly efficient production processes. The bulk production of parts and components in the automotive, construction, energy, house-hold appliance, and related industries necessitates the use of finishing techniques for parts on a mass scale. The solutions to the problem of ensuring high quality surfaces in the manufacturing process are different. The studies on advanced tools [1,2], increasing processing parameters [3,4,5] as well as automation of processes should be mentioned in this context. One of the highly efficient methods of finishing is vibro-abrasive processing [6]. Vibro-abrasive processes provide a wide range of possibilities for finishing edges and surfaces of produced elements [7]. Processing with the use of loose abrasive material has found its application beginning from simple deburring processes to advanced solvent polishing or strengthening of the workpiece surface [8]. The vibro-abrasive technique can be used to process various materials, from plastics through non-ferrous metal alloys, to steel parts. Nowadays, a complicated shape of the workpiece and its size determine only the selection of the appropriate equipment and abrasive media. Continuous progress in the improvement of various types of abrasive media and auxiliary chemical compounds is the reason for continuous optimization of the process and its parametrization [9,10,11].

Aluminium alloys belong to a group of materials which has stayed in many branches of industry for good. Due to their low weight and good strength, aluminium alloys compete effectively with steels in the automotive industry. Vibratory finishing processes have found their applications starting from small parts made of casting alloys, through large bodies, to forged elements made of plastically formed aluminium alloys [12,13,14,15,16].

Machine parts subjected to vibratory processing, the purpose of which is to polish their surface, each time require preliminary preparation of the surface. Vibro-abrasive high gloss finishing of aluminium alloy work-pieces most often takes place in three successive stages: grinding, lapping and polishing. The first stage, the so-called vibratory grinding, is the stage where the burr from the production process is removed, sharp edges are rounded off and, above all, the surface is unified through reducing its roughness to the greatest possible extent. Appropriate parametrization of the grinding stage, i.e., the selection of abrasives, auxiliary chemicals and processing time have an effect on the performance and overall time of the entire process. The wide variety of abrasive media and machine types used for such processing means that every part requires an individual approach.

In this paper, an attempt is made to parametrize the vibratory grinding process, which is one of the three stages of high gloss finishing. Samples of the 6082 aluminium alloy intended for use in loaded machine parts and forged car wheel rims were subject to a research analysis. The samples were processed in a rotary vibrator equipped with a sample fixing system, using resin media and auxiliary compounds. On the basis of the analysis, the processing capacities were determined for the selected conditions and abrasive media. The presented research results can be considered as complementary to the knowledge of the possibilities of the described process and, to some extent, they are a continuation of similar works [7,8,17]. It should be added that the vibro-abrasive processes, due to the wide range of their possible applications, still require research and deepening of the knowledge in this area.

## 2. Research Material

The material for the research consisted of samples made of 5-mm thick sheet aluminium alloy of grade 6082. Properties of the material to be tested, as specified by standards, and equivalent symbols for selected standards are presented in Table 1.

**Figure 1:**
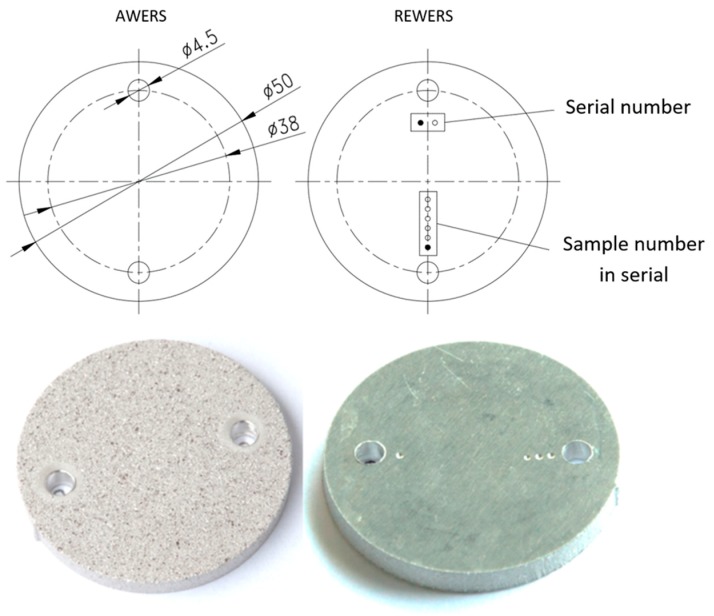
Geometrical characteristics of the samples and the applied system of sample designation.

The samples were cut out using the water-jet technique from previously shot-blasted pieces. The purpose of shot blasting was to give a uniform initial surface roughness for the Ra index above 6.3 µm, and for Rz above 25 µm. The assumed range of initial roughness corresponds to the most frequently obtained surfaces of parts produced through casting, plastic processing, or coarse machining. The geometrical characteristics of the samples and the applied designation system are presented in Figure 1.

## 3. Research Equipment and Methodology

**Figure 2:**
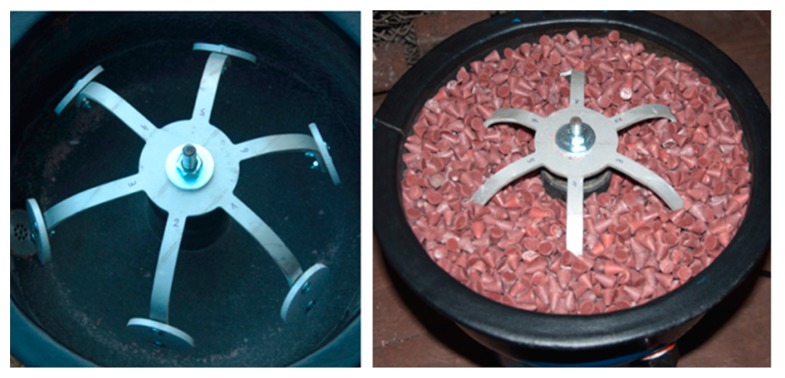

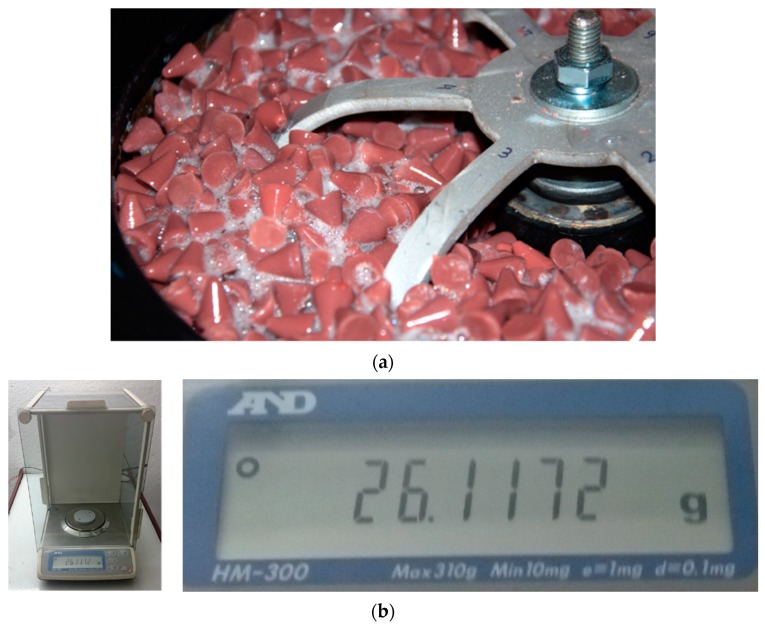
(**a**) The test rotary vibrator with the mounted sample fixing system and samples; (**b**) Measuring scales and its parameters.

Tests were performed using a rotary vibrator with 8 dm^3^ nominal capacity, fitted with a sample fixing system and a flushing system of the work bowl. The test rotary vibrator was equipped with a classic 1450 rpm vibratory drive generating 4–6 mm amplitude vibrations. The device together with the mounted sample fixing system and filled with abrasive media is presented in Figure 2a. The abrasive media used for processing were resinoid-bonded abrasives with the S12TZ medium processing intensity, intended for preliminary processing, grinding and deburring of workpieces before their lapping and polishing. The process was carried out with the use of the RO-ALU-1 auxiliary compound in a concentration of 3%, at a rinsing rate of 5 dm^3^/h. Appropriately marked samples were removed one by one from the work bowl every 2 h. Each of them was cleaned in an ultrasonic washer, dried, and weighed both before and after processing. The processed samples were subjected to surface roughness tests. The roughness of the reference sample after shot blasting was adopted as the reference surface condition.

Surface roughness measurement was performed using the FRT-Micro Prof100 profilometer with a CWL600 head, with the capacity to measure 2D and 3D surfaces in the area of 10 × 2.5mm. The roughness was measured in the central part of the sample which had direct contact with the media during processing. A precision balance of A&D Company, model HM-300, was used to measure the mass of the samples. The measuring scales and its precision (0.0001 g) are presented in Figure 2b.

## 4. Research Results

In order to illustrate the effect of vibro-abrasive processing on the surface roughness of the 6082 alloy specimens, a comparative analysis was performed in relation to the reference (primary) sample after shot blasting. The reference sample was not subjected to vibratory processing. Photographs of the samples after vibro-abrasive processing, weight and roughness measurement results, and 3D and 2D surface analysis results are juxtaposed in Table 2, Table 3, Table 4, Table 5, Table 6, Table 7 and Table 8. The compiled results cover the range of 12 h of vibro-abrasive processing under the assumed operating conditions.

## 5. Analysis and Discussion of Research Results

**Figure 3:**
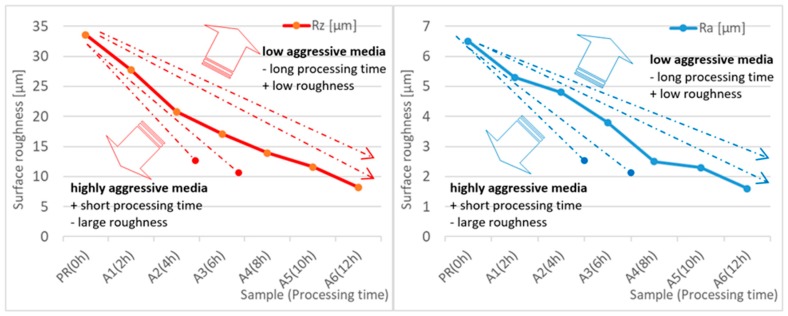
Graphical juxtaposition of changes in roughness parameters for the tested samples and the estimated effect of different types of abrasive media on surface roughness and processing time.

The main parameters, on the basis of which the effect of the applied vibratory processing on the quality of the obtained surfaces can be illustrated are, first of all, the roughness parameters, Ra, Rz, and weight loss of the workpiece. The arithmetic mean deviation of the rough-ness profile Ra and the maximum height of the roughness profile Rz (according to ISO 4287) are the most common values in industrial practice. These parameters have permanently entered the technical documentation as well as a wide range of surface quality templates, which are used by designers, process engineers and operators directly performing the required process on the workpiece. Analyzing the test results for selected abrasive media, a reduction in surface roughness from 6.5 µm to 1.6 µm for the Ra index, and from 33.6 µm to 8.2 µm for the Rz index can be observed. Taking into consideration the 12-hour processing time, it can be assumed that the hourly reduction in surface roughness for Ra is about 0.46 µm and for Rz, about 2.40 µm. The weight loss of the sample (work-piece) is additional information and is used mainly by process engineering to select a suitable processing material. The weight loss is a value that characterizes the processing intensity over time. In the case under consideration, the average hourly weight loss of the samples can be estimated at 0.024 g. Taking into account the results of tests performed on samples made of the 6082 aluminium alloy, a numerical and graphical juxtaposition of the analyzed surface roughness parameters is presented in Table 9 and in Figure 3.

One of the factors influencing the change of qualitative and quantitative parameters of samples during vibratory processing is the selection of abrasive media with appropriate shape and appropriate abrasive parameters (aggressiveness). The selection of abrasive media also has a great influence on the processing time. When selecting appropriate processing parameters, preliminary (exploratory) tests are often conducted in order to make a preliminary assessment of the influence of the selected parameters of vibratory processing on the obtained quality parameters of the material processed. Since the expected quality of vibro-abrasive processing is influenced by a number of variables, preliminary tests are often carried out multiple times [19,20].

Within the framework of the authors’ own research, an analysis was made to assess the possibility of replacing the applied media with solutions representing other—higher or lower—abrasive parameters (aggressiveness) and their influence on the quality parameters of samples made of the 6082 aluminium alloy. There are additional lines plotted in Figure 3 which present the possible influence of other types of abrasive media on the processing time and the possible surface roughness which can be obtained through vibratory processing. The use of samples with lower abrasive parameters increases the experiment time, but it offers the possibility to obtain surfaces with higher quality parameters. Higher aggressive parameters of abrasive media reduce processing time but may result in the failure to achieve the required Ra and Rz parameters during vibratory processing, despite the permissible mass loss of the workpiece. As results from the study, the experiments conducted on S12TZ media are characterized by a medium level of abrasiveness.

**Figure 4:**
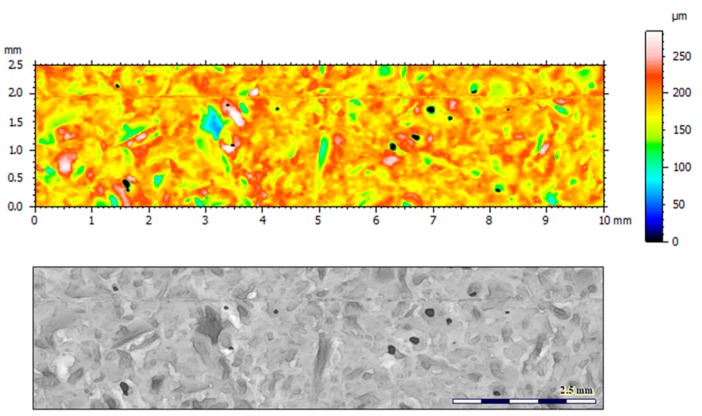
2D isometric map and numerical photo of the surface of the PR sample (before vibro-abrasive processing).

**Figure 5:**
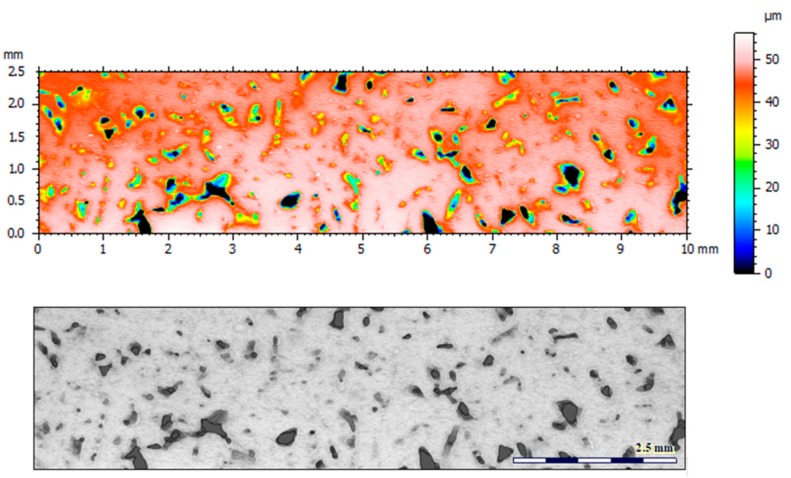
2D isometric map and numerical photo of the surface of the A6 sample (after vibro-abrasive processing).

Figure 4 and Figure 5 present examples of the geometrical shape of the tested surface before and after vibro-abrasive processing. Further, 2D isometric maps and numerical photographs of sample surfaces are presented. When observing the surface topography and the assigned colour scale before and after vibro-abrasive processing, one can see more than a five-fold reduction in roughness. The threshold local roughness peaks were considerably reduced from more than 250 μm to about 50 μm. The qualitative changes visible in Figure 4 and Figure 5 confirm the correctness of the selection of vibratory processing parameters in the experiments carried out in the study.

**Figure 6:**
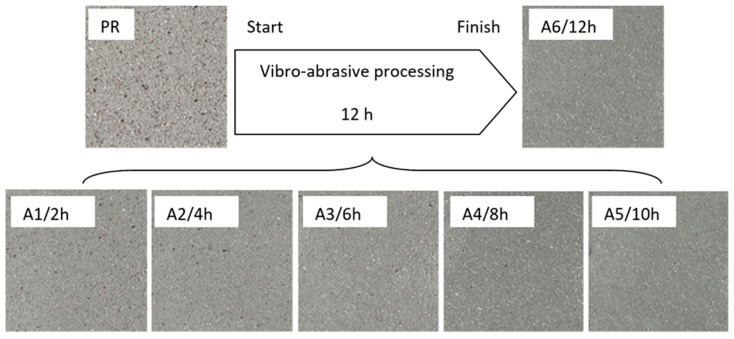
Photographs of surfaces of the tested samples.

In order to further evaluate the surface quality of the analyzed 6082 aluminium alloy samples, enlarged photographs of the surfaces after successive processing phases are presented in Figure 6. The surface changes observed in this case may be an additional comparative parameter in the quality assessment of the processed surfaces. Such a comparison can also be used as a template of surface roughness of aluminium alloy 6082 for the subsequent processing times where the analyzed S12TZ abrasive media are used.

**Figure 7:**
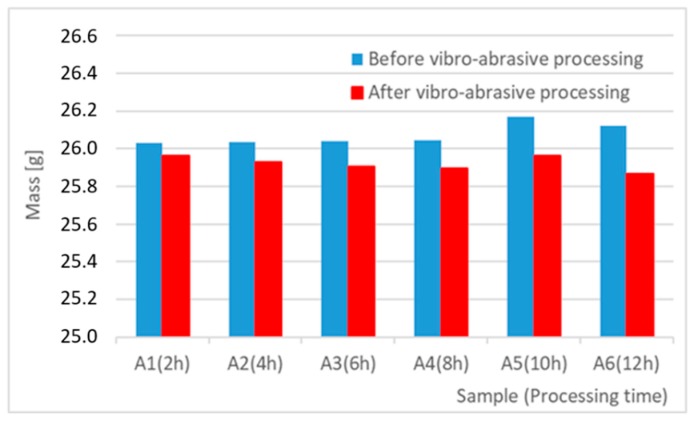
Comparison of sample weights before and after vibro-abrasive processing.

**Figure 8:**
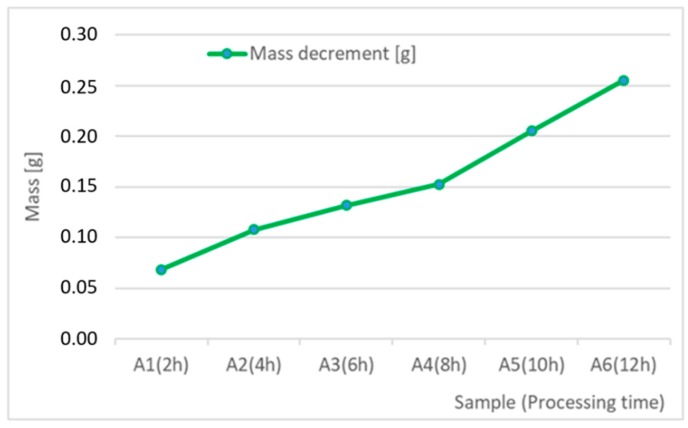
Sample weight loss as a function of vibro-abrasive processing time.

Table 10 presents the results of measurement of sample weights before and after vibro-abrasive processing and sample weight loss as a function of processing time. Additionally, Figure 7 and Figure 8 present the results in a graphic form together with their linear averaging.

The results of the research show that reducing, during a 12-hour experiment, the roughness of the 6082 aluminium alloy samples from approx. 250 µm to approx. 50 µm (Figure 4 and Figure 5) caused a maximum reduction in the mass of the samples below 1%. Such an insignificant reduction in mass indicates the correct choice of S12TZ abrasive media and vibro-abrasive processing parameters (Section 3), since the contact interaction of the media resulted in the removal of roughness peaks in the samples without affecting the other surfaces in a too aggressive manner.

## 6. Conclusions

Based on the tests performed, the effect was determined of the time of vibro-abrasive processing with the use of selected abrasive media and compounds on changes in surface roughness and weight loss of the samples. The tested material belongs to the group of aluminium alloys which is very often used in the production of forged aluminium wheel rims. Due to the developing technology of vibratory high gloss finishing of these vehicle components, the first stage of vibratory grinding is the longest and requires a proper selection of abrasive media. The correct parametrization of the process and correct selection of processing materials have a direct influence on the processing time and quality of the obtained surface roughness indices. It should be remembered that the vibratory grinding process is the first phase and its outcome affects the time of the subsequent phases, i.e., smoothing and solvent polishing. Proper selection of processing media in the most time-consuming stages of processing, always has an impact on the overall cost of the entire process. The study has shown that the use of S12TZ type resin media for vibro-abrasive processing of samples made of the 6082 aluminium alloy enables reducing the rough-ness by 75.5% in a 12-hour work cycle. This value allows us to assume an hourly improvement in surface quality of 6.3%. It should be mentioned that the significant development of processing media and media supporting vibratory processing offers greater and greater possibilities. This creates a huge space for analyses and the search for optimal processing parameters for selected machine parts.

## Figures and Tables

**Table 1:** Characteristics of alloy 6082 [18].

Designation			
PN/EN	DIN	ISO	Werkstoff
6082	AlMgSi1	AlSi1MgMn	3.2315
Chemical composition (%) as per EN 573-1			
Mg	0.60–1.20	Zn	≤0.20
Mn	0.40–1.00	Cr	≤0.25
Fe	≤0.50	Ti	≤0.10
Si	0.70–1.30	other	≤0.15
Cu	≤0.10	Al	Rest
Mechanical properties of smooth steel sheets for thicknesses of 3–6 mm in the T6-strengthening state according to EN 485-2			
Rm min	310 MPa	A min	10%
Rp0, 2 min	260 MPa	Hardness	94 HB

**Table 2:** Compiled test results for the reference sample PR.

Reference Sample, PR	
Sample after shot blasting/without vibratory processing	
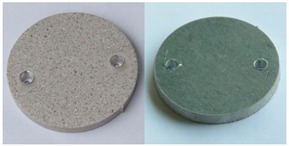	
Sample weight before vibratory processing:	26.0356 g
Sample weight after vibratory processing:	-
Difference in weight before and after processing:	-
3D image of sample surface	
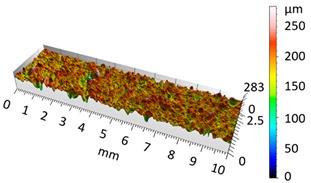	
2D profile of the surface examined	
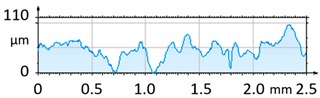	
Sample roughness, Ra	6.5 µm
Sample roughness, Rz	33.6 µm

**Table 3:** Compiled test results for sample A1.

Sample A1	
Sample after vibratory processing/processing time: 2 h	
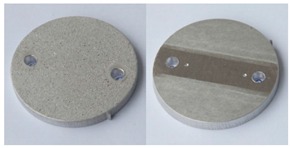	
Sample weight before vibratory processing:	26.0313 g
Sample weight after vibratory processing:	25.9627 g
Difference in weight before and after processing:	0.0686 g
3D image of sample surface	
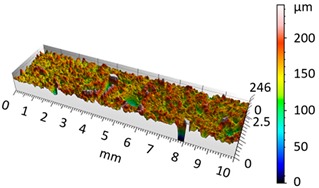	
2D profile of the surface examined	
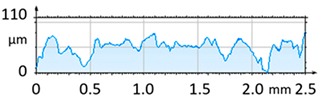	
Sample roughness, Ra	5.4 µm
Sample roughness, Rz	27.7 µm

**Table 4:** Compiled test results for sample A2.

Sample A2	
Sample after vibratory processing/processing time: 4 h	
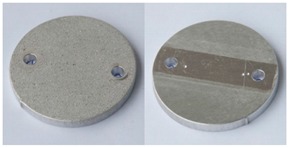	
Sample weight before vibratory processing:	26.0353 g
Sample weight after vibratory processing:	25.9275 g
Difference in weight before and after processing:	0.1078 g
3D image of sample surface	
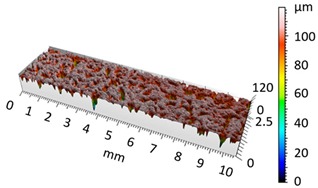	
2D profile of the surface examined	
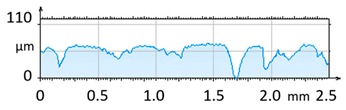	
Sample roughness, Ra	4.8 µm
Sample roughness, Rz	20.8 µm

**Table 5:** Compiled test results for sample A3.

Sample A3	
Sample after vibratory processing/processing time: 6 h	
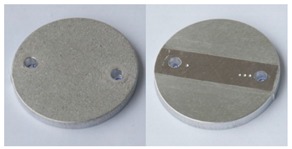	
Sample weight before vibratory processing:	26.0370 g
Sample weight after vibratory processing:	25.9052 g
Difference in weight before and after processing:	0.1318 g
3D image of sample surface	
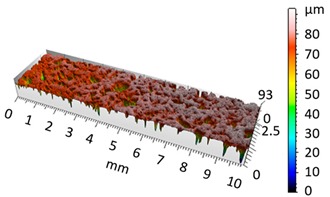	
2D profile of the surface examined	
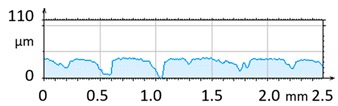	
Sample roughness, Ra	3.8 µm
Sample roughness, Rz	17.1 µm

**Table 6:** Compiled test results for sample A4.

Sample A4	
Sample after vibratory processing/processing time: 8 h	
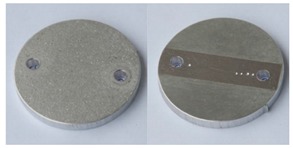	
Sample weight before vibratory processing:	26.0462 g
Sample weight after vibratory processing:	25.8937 g
Difference in weight before and after processing:	0.1525 g
3D image of sample surface	
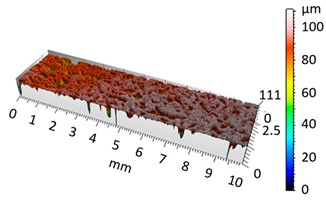	
2D profile of the surface examined	
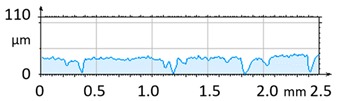	
Sample roughness, Ra	2.5 µm
Sample roughness, Rz	13.9 µm

**Table 7:** Compiled test results for sample A5.

Sample A5	
Sample after vibratory processing/processing time: 10 h	
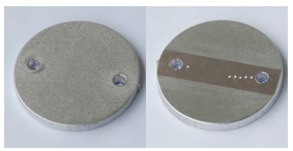	
Sample weight before vibratory processing:	26.1678 g
Sample weight after vibratory processing:	25.9623 g
Difference in weight before and after processing:	0.2055 g
3D image of sample surface	
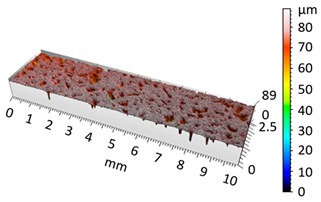	
2D profile of the surface examined	
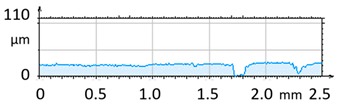	
Sample roughness, Ra	2.3 µm
Sample roughness, Rz	11.6 µm

**Table 8:** Compiled test results for sample A6.

Sample A6	
Sample after vibratory processing/processing time: 12 h	
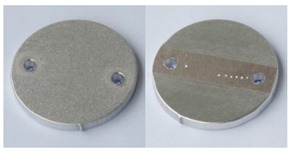	
Sample weight before vibratory processing:	26.1198 g
Sample weight after vibratory processing:	25.8644 g
Difference in weight before and after processing:	0.2554 g
3D image of sample surface	
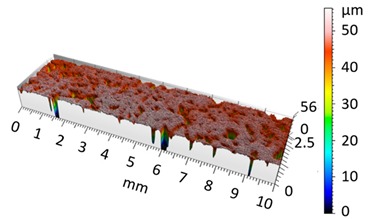	
2D profile of the surface examined	
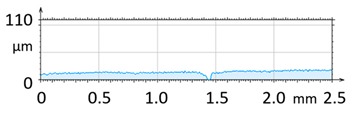	
Sample roughness, Ra	1.6 µm
Sample roughness, Rz	8.2 µm

**Table 9:** Numerical juxtaposition of roughness parameters.

Sample Number	Surface Roughness		Processing Time
	Ra (µm)	Rz (µm)	(h)
**PR**	6.5	33.6	0
**A1**	5.3	27.7	2
**A2**	4.8	20.8	4
**A3**	3.8	17.1	6
**A4**	2.5	13.9	8
**A5**	2.3	11.6	10
**A6**	1.6	8.2	12

**Table 10:** Juxtaposition of the results of sample weight measurements before and after vibro-abrasive processing, and sample weight loss as a function of time.

Sample Number	Mass (g)		Mass Decrement (g)	Processing Time (h)
	Before Vibro-Abrasive Processing	After Vibro-Abrasive Processing		
**A1**	26.0313	25.9627	0.0686	2
**A2**	26.0353	25.9275	0.1078	4
**A3**	26.0370	25.9052	0.1318	6
**A4**	26.0462	25.8937	0.1525	8
**A5**	26.1678	25.9623	0.2055	10
**A6**	26.1198	25.8644	0.2554	12
